# Annotations

**Published:** 1903-09-26

**Authors:** 


### Annotations.

### Boys' Camps.

Not many forms of philanthropy can claim to do more for the moral as well as the physical health of those whom they assist than boys' seaside and country camps. It is not merely that the boys get fresh air. The great merit of the camp life is that it gives the maximum of fresh air with the minimum of supervision. The life teaches self-reliance and self-respect. If a boy is bright and active he has scope for his energy without getting into really dangerous or unwholesome mischief. A boy who has any initiative finds plenty of room to exercise it in the chances of camp life, where wind and weather, to mention no other accidents, prevent a monotonous sameness in the day's routine. Ideas which might never dawn in his brain in town, are found to be useful and practical in the new environment. And the boy who is rather soft and sickly will be braced, not only by the keen air, but by the inevitable "roughing it" of life under canvas, while at the same time any feminine accomplishments he may possess in the way of cooking and washing-up will prove so useful to the community that they will win him respect and not contempt, as they might in other circumstances. Town life at the best is hedged in by observances, and organisation hinders observation. The stories of cockney ignorance of country life may be exaggerated, but they have a fundamental truth. A town child does not actually and consciously believe that milk is native to a tin pail or that a whiting goes through life with its tail stuck through its eye-sockets, but being familiar with these and all other commodities only in a more or less ready-made condition tends to an indefinable levity of thought. Life is too easy for the townsman—even in the slums. When he has once seen, or, even better, attempted the operation of milking a restless cow, or hopefully, though fruitlessly, dangled a baited line, he will realise the scheme of civilisation as well as of nature better than he could by means of a hundred lectures. It will all be part of the fun, no doubt, but it will also be part of an education that will impress on him that gospel of labour which it is the instinctive desire of humanity, and especially of youthful humanity, to forget. A fortnight's camping out will send a boy home not only stronger in wind and limb, but possessed of a larger and saner outlook on life which will make him, even in the city, a better and a wiser man.

### Eye-Strain and General Health.

A well-known physician from the other side of the Atlantic has, with true American energy, set himself to establish the doctrine that for many of the pains and pangs endured by mortal flesh it is "eye-strain" which must be held responsible. More particularly, it is urged, is this true of the complaints and sufferings of genius. The habits of De Quincey, the crustiness of Carlyle, the dyspepsia of Darwin, the hypochondria of Huxley, the shattered nerves of Spenser, are all alike to be referred to one and the same fundamental misfortune. Errors of refraction, uncorrected—it is these which have made so many men to suffer, not always in silence. It must be allowed that at the first blush this seems, if the expression may be permitted, a somewhat "tall order." It suggests itself as one more instance of the common passion for a universal panacea and as an outcome of the habit of looking at the processes of disease from a special and narrow point of view. Like the rest of us, Dr. Gould may on the basis of his own therapeutic successes be too ready to call upon everyone to see the world through his special spectacles. But a study of his writings will compel the most incredulous to admit that he exhibits great diligence and ingenuity in the defence of his thesis. Indeed, it would be difficult to deny in the face of the evidence submitted that there must be some truth in the suggestion. The question is certainly one worthy of careful study. Apart from the possibility of relieving much suffering, there is in Dr. Gould's teaching a wholesome influence which may help to correct certain unfortunate modern tendencies. His studies are based upon the contemplation not of any one part or organ, but include the entire man. They recognise that the organism is a unit with interdependent parts, and that disease may be functional and apart from organic change. Whatever emphasises these two old-fashioned truths is an influence in favour of sound practice alike in diagnosis and treatment.

### The Certificate Nuisance.

It is only too certain that the medical profession permits itself to be systematically exploited for the benefit of organisations which have no claim on its services. A growing illustration of this is to be found in the certificate nuisance. Not merely private clubs and benefit assurance societies, but various public bodies are now on a large scale exacting certificates from medical practitioners without any recognition or fee. The arrangement by which this end is secured is as simple as it is ingenious. The absence of the certificate is made to carry certain disabilities to the patient, it may be the refusal of a weekly allowance, or exclusion from the benefits of a convalescent home, or a summons to appear before a magistrate because of a child's absence from school. An appeal *ad misericordiam* is therefore easily stated. To this it appears ungracious for the practitioner to turn a deaf ear, and he therefore fills in and signs the certificate, and thus adds one more to many other influences by which the value of medical advice and responsibility is depreciated in the public judgment. Many of the certificates submitted in these circumstances are quite elaborate documents. So many particulars as to the stage, progress, etc., of the disease are demanded, that a very considerable amount of time and trouble is involved. If the details are neglected or refused, it is open to some junior office clerk to decline the certificate and thus to inflict inconvenience or even suffering on the patient. It is high time that the profession made a stand against this growing practice. If the organisations demanding these certificates cannot carry out their schemes without them they ought to meet the necessary expense in the usual way and not put the medical profession under tribute. Moreover, it is in the public interest to have professional confidences held inviolate, and a practice which outrages this ought to be met with steady opposition.

